# The effect of 1,2-dioleoyl-3-trimethylammonium propane (DOTAP) Addition on the physical characteristics of β-ionone liposomes

**DOI:** 10.1038/s41598-023-31560-5

**Published:** 2023-03-15

**Authors:** Andang Miatmoko, Febe Harum Asmoro, Andre Alwi Azhari, Noorma Rosita, Chin-Shiu Huang

**Affiliations:** 1 Department of Pharmaceutical Sciences, Faculty of Pharmacy, Universitas Airlangga, Campus C UNAIR, Surabaya, 60115 Indonesia; 2 Stem Cell Research and Development Center, Universitas Airlangga, Campus C UNAIR, Surabaya, 60115 Indonesia; 3 Department of Food Nutrition and Health Biotechnology, Asia University, Liofang Road, Wufeng District, Taichung, 413545 Taiwan

**Keywords:** Materials science, Nanoscience and technology

## Abstract

β-ionone (ION) is a cyclic terpenoid compound that demonstrates considerable potential for the prevention and treatment of cancer. However, the water solubility of β-ionone is poor and the compound demonstrates low permeability. Liposomes have been reported as increasing both qualities. In this study, the development of β-ionone liposomes was initiated by adding 1,2-dioleoyl-3-trimethylammonium propane (DOTAP) to produce cationic liposomes as a means of enhancing binding to cancer cells. Liposomes composed of β-ionone, HSPC, cholesterol, and DSPE-mPEG_2000_ were prepared using the thin layer hydration method. Cellular uptake studies were carried out with HeLa cells incubated with β-ionone liposomes for two hours. The results indicated that the addition of DOTAP increased particle size and affected the spectroscopical and thermogram profiles of the liposomes, thereby confirming reduction in liposome crystallinity, while the zeta potential became positive. Moreover, the calcein release profile further showed that additional DOTAP increased both membrane fluidity and cellular uptake in HeLa cells In conclusion, adding DOTAP affected the physicochemical cationic properties of liposome and improved cellular uptake in HeLa cells.

## Introduction

β-ionone is a cyclic terpenoid compound with a basic core structure of retinoic acid, retinol, -carotene, and vitamin A which is found in many fruits, vegetables, and grains such as carrots, raspberries, almonds, tea, and tomatoes^1^. Research has shown that β-ionone has the potential to both prevent and treat several types of cancer, such as breast, colon, lung, and liver^2^. The anticancer activity involves several mechanisms, including antiproliferation, where β-ionone inhibits the proliferation of lung cancer cells (A549) by inducing apoptosis as seen from the increased expression of the Bax gene and reduced expression of the Bcl-2 gene which suppresses the expression of cPLA2 and COX-2 proteins^3^. β-ionone causes apoptosis, as seen in lung cancer (A549) cell death, (A549) through loss of mitochondrial membrane potential as measured by staining using rhodamine-123^4^. Besides that, β-Ionone can inhibit the in vitro proliferation of breast cancer cells. One of the mechanisms for inhibiting MCF-7 cell proliferation is reducing cyclooxygenase (COX-2) activity in the induction of apoptosis of MCF-7 cells. β-Ionone at a concentration of less than 50 µmol/L can reduce the expression of COX-2 protein in mice tumor tissue, which is considered a target for cancer molecules^5^.

Dissolving β-ionone in water at a level of 0.169 g/L is extremely difficult. β-ionone also has low permeability with log *P* = 4^6,7^ and is classified as falling within Biopharmaceuticals Classification System (BCS) class IV. Numerous studies have shown that using liposomes can improve the solubility and permeability of compounds belonging to class IV BCS^8^.

Liposomes are lipid vesicles consisting of an aqueous phase core surrounded by one or more phospholipid bilayers^9^. Liposome membranes are composed of natural and/or synthetic phospholipids which are relatively biocompatible, biodegradable, and non-immunogenic. Due to the unique bilayer structure, liposomes act as carriers for both lipophilic and hydrophilic materials. The latter materials are in the aqueous phase, while the former are trapped in the lipid bilayer^10^.

The liposome delivery system is reported as overcoming Lopinavir's low solubility and bioavailability to increase therapeutic efficacy^11^. The use of liposomes can also increase drug permeability. The particle size of liposomes allows higher intracellular uptake than other systems, thereby increasing drug bioavailability. Previous research has indicated that liposome encapsulation can significantly increase macrovascular permeability after administration of Paclitaxel liposomes^12^, where Paclitaxel is also classified as the compound in BCS class 4, like β-ionone.

The nature and charge density of liposomes affect the stability, kinetics, and extent of biodistribution and interactions with liposome uptake by target cells^13^. Cationic liposomes have the advantage of promoting higher therapeutic efficacy against cancer cells^9^, where they can bind to negatively charged cell membranes. Other studies have shown that they accumulate selectively in tumor angiogenic endothelial cells and are superior to anionic or neutral liposomes^14,15^. The research results obtained by Peters et al. (2015) showed that the cell uptake of 1,2-Dioleoyl-3-trimethylammonium propane (DOTAP)-liposomes was higher than that of the 1,2-dioleoyl-L-a-glycero-3-phosphatidylethanolamine (DOPE)-liposomal formulation. Another study using DOTAP as lipid composition demonstrated that transgene expression was increased in tumor cells due to the uptake of liposome-DNA complexes by tumor cell phagocytosis in vitro^16^. Research conducted on the cytotoxicity test confirmed that adding DOTAP to liposomes increased the cytotoxicity of HepG2 cancer therapy^17^.

Cationic lipid^17^ strongly influences biodistribution and drug efficiency^17,18^. DOTAP is a phospholipid type frequently employed as a component of cationic liposomes. DOTAP contains an NH^4+^ group which renders it positively charged and is low in toxicity compared to other cationic lipids^19^. Based on the research of Chang et al. (2018), the addition of DOTAP to the liposome formulation causes a change in size^20^. The liposomes prepared with PC only have a larger size than those prepared with a 1:1 ratio of PC and DOTAP (PC/DOTAP liposomes) with the same amount of lipids. This is because by accumulating the same amount of lipids, DOTAP, which has a shorter lipid chain, reduces the size of the liposome particle^21^. In the DOTAP-DNA complexes, the charge ratio of lipid/DNA highly affects the physical properties of the liposome. The addition of low-concentration DOTAP produced a larger size, namely 190 ± 8.4 nm, compared to liposomes with a high concentration of DOTAP, which was 140 ± 3.8 nm^22^. DOTAP-induced changes at the membrane interface may facilitate electrostatic interactions in the polar head region. This physical interaction can decrease the particle size by reducing the radius of bilayer curvature^23^. In addition, the low lipid/DNA charge ratio enhanced the repulsion of excess negative charges, resulting in small particle sizes.

The relationship between liposome vesicle size and cell interactions was observed by means of the uptake from bone marrow-derived macrophages (BMDM). The absorption of BMDM from small DOTAP: DOPE liposomes was superior to that of their larger counterparts. Small cationic liposomes tend to be distributed to lymph nodes more rapidly from the injection site than larger ones. The liposome charge was also observed where the addition of DOTAP as a cationic lipid resulted in an extremely positive zeta potential of approximately + 40 mV to + 60 mV^24^.

It was additionally reported that the dispersibility of liposomes following the addition of DOTAP was more effective than without DOTAP due to the presence of electrostatic forces^25^. Furthermore, another study stated that adding DOTAP can render liposomes spherical with an approximate size of 100 nm^15^. On the other hand, adding DOTAP can increase the fluidity of the bilayer membrane. This is evidenced by the research of Takechi-Haraya et al. (2016), where DOTAP/Chol (90/10) has a liquid disorder arrangement, while DOTAP/Chol (50/50) has a liquid order arrangement which was observed using the Atomic Force Microscope (AFM). It is also proven by the deformation force curve where DOTAP/Chol (50/50) is steeper than DOTAP/Chol (90/10)^26^.

Lipophilic compounds such as β-ionone will be entrapped in the liposome membrane which will, in turn, affect its fluidity. The results showed that the greater the amount of β-ionone trapped, the faster the release rate^27^. Previous studies have also reported that drug compounds can interact with phospholipids, thereby affecting membrane fluidity^28–32^. Therefore, the membrane's fluidity will affect the stability of the drug entrapment in liposomes and the drug released during circulation, thus determining the effectiveness of the treatment^28^. Therefore, it is necessary to test the fluidity of the membrane, one method being calcein release. Calcein is a water-soluble, quenching, and fluorescent substance widely used in cell viability studies^33^. Calcein release studies can be undertaken to determine membrane stability related to the presence or absence of liposome leakage measured using a spectrofluorometer^34^.

In this study, the effect of the addition of different levels of DOTAP on the membrane's physical characteristics and the conducting of a fluidity test were evaluated. The data obtained may yield information on the effect of adding DOTAP to β-ionone liposomes and the development of further liposome formulations for inclusion in anticancer therapy.

## Result

### The particle size and zeta potential of β-ionone liposomes

**Figure 1:**
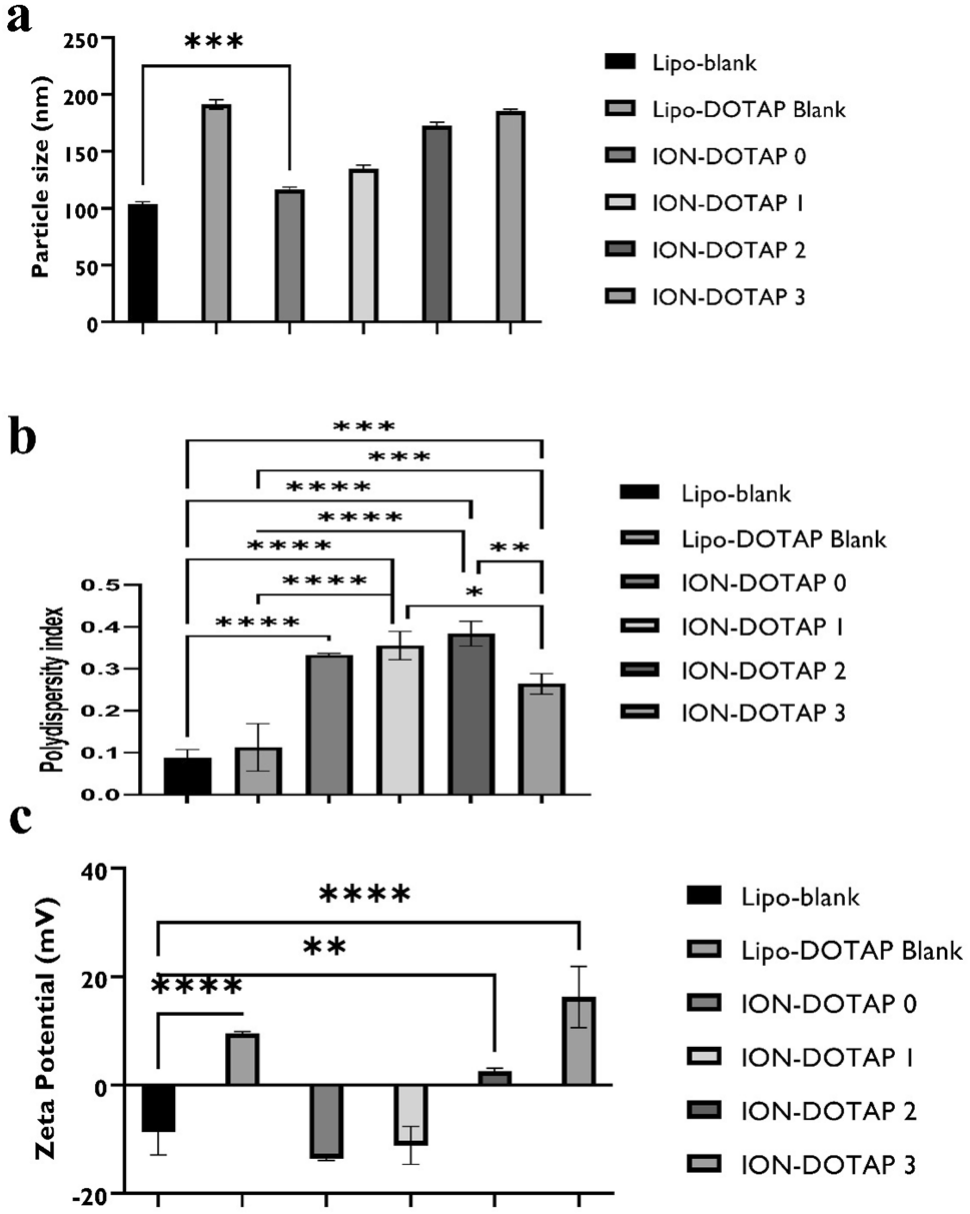
The bar charts of average (**a**) particle size value, (**b**) polydispersity index and (**c**) zeta potential of β-ionone liposomes prepared with various concentrations of DOTAP. Lipo-Blank contained no DOTAP or β-ionone, while Lipo-DOTAP Blank contained DOTAP at the highest concentration without the addition of β-ionone, *p < 0.5, **p < 0.01, ***p < 0.001, compared to Lipo-Blank.

The addition of DOTAP concentration tended to increase particle size. The largest liposome with an average size of 191.27 ± 4.20 nm was achieved by lipo-DOTAP blank. However, the size increased with the addition of DOTAP to the formula, as shown in Fig. 1a. PDI measurements were carried out to determine the homogeneity of particle size. Particles with smaller PDI values indicate a system with a homogeneous size distribution. PDI values > 0.7 indicate that the preparation has an extensive distribution of particle sizes^35^. The lowest PDI value was obtained by the Liposome Blank formula, as shown in Fig. 1b.

For the zeta potential value, the results indicated that the Lipo-blank, ION-DOTAP-2, and ION-DOTAP-3 liposomes differed significantly from liposomes ION-DOTAP-0, while Lipo-blank and ION- DOTAP-1 did not show any difference with ION-DOTAP-0 as shown in Fig. 1c.

### The vesicle morphology of β-ionone liposomes analyzed by transmission electron microscopy (TEM)

**Figure 2:**
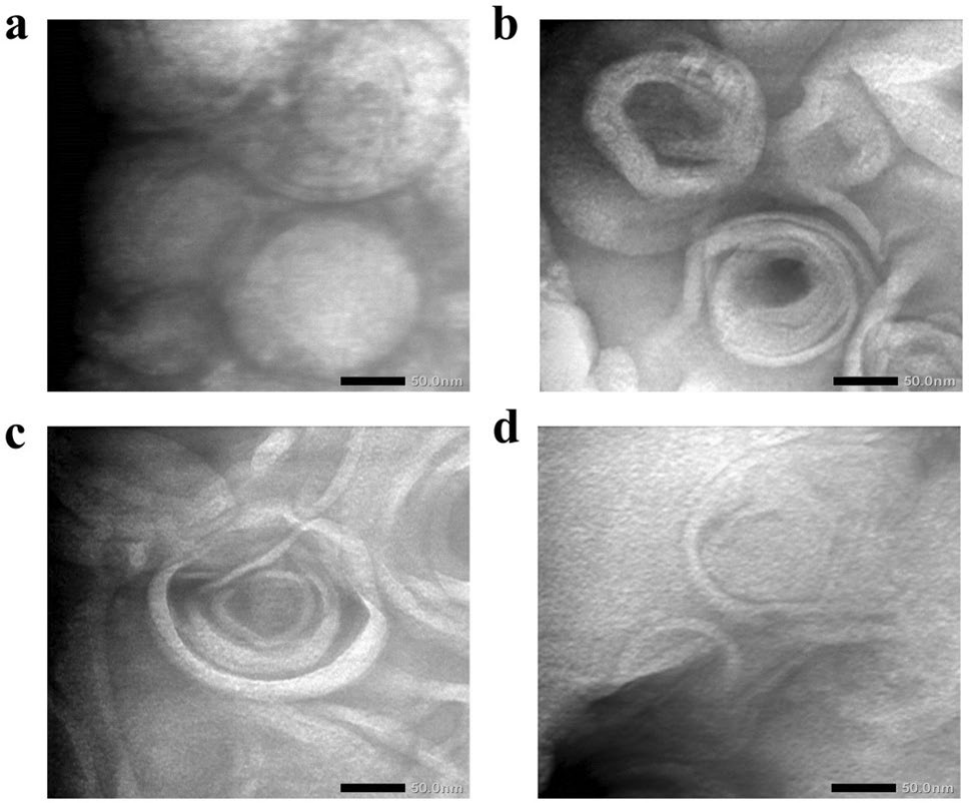
Morphological observations of (**a**) ION-DOTAP-0, (**b**) ION-DOTAP-1, (**c**) ION DOTAP-2, and (**d**) ION-DOTAP-3 liposomes were analyzed using Transmission Electron Microscopy (scale bar: 50 nm).

From the results of the vesicle morphology analysis with TEM contained in Fig. 2, it can be observed that the vesicles of ION-DOTAP-0 liposomes appear spherical with unilamellar membrane formation. In contrast, the formula, while also apparently spherical in shape, has a layered membrane due to the addition of DOTAP.

### The spectra profiles of fourier transform infrared spectroscopy (ftir) of β-ionone liposomes

**Figure 3:**
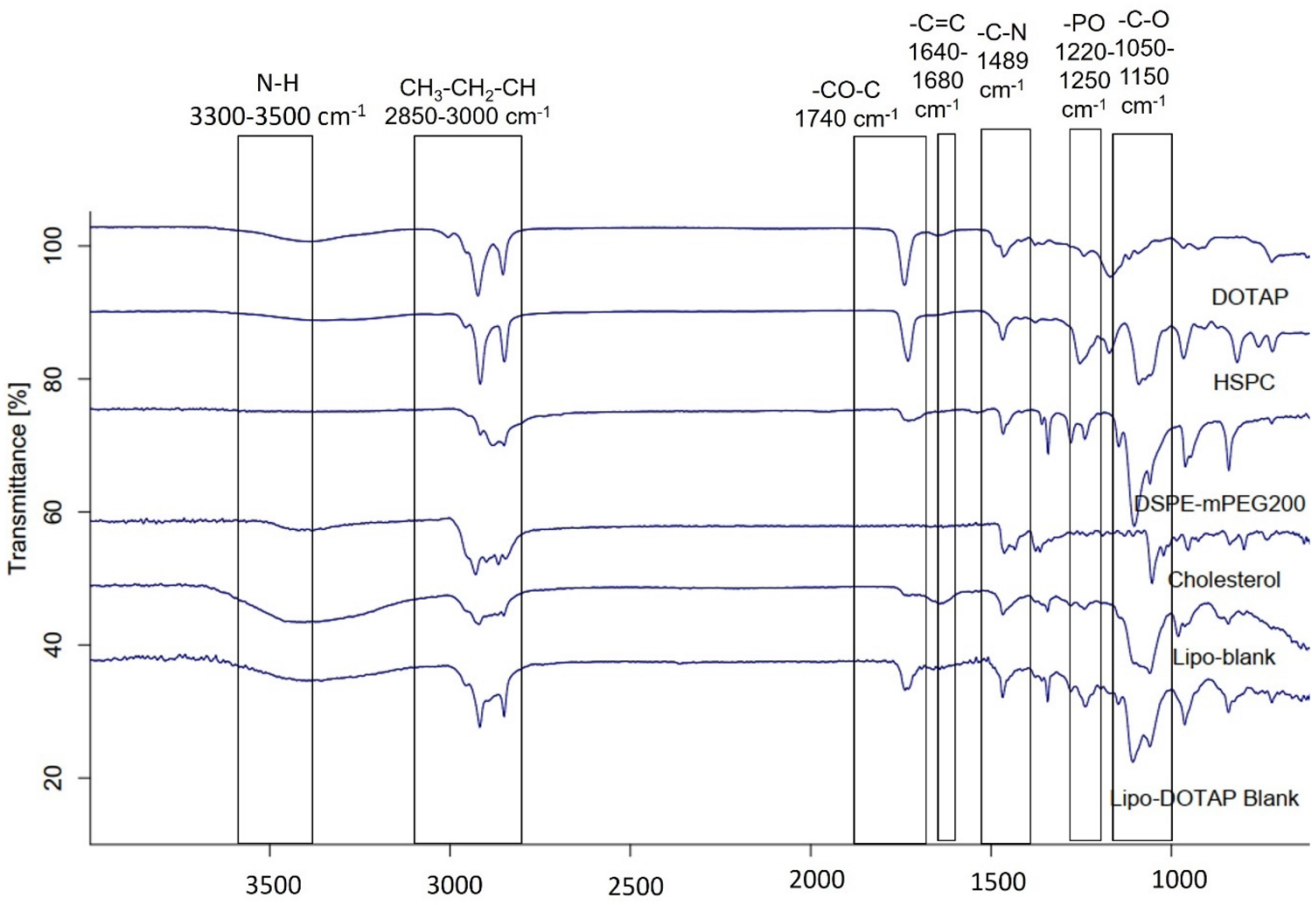
FTIR spectra of β-ionone, DOTAP, HSPC, DSPE-mPEG2000, cholesterol, Lipo-Blank, and Lipo-DOTAP Blank. Lipo-Blank contained no DOTAP or β-ionone, while Lipo-DOTAP Blank contained DOTAP at the highest concentration without the addition of β-ionone.

**Figure 4:**
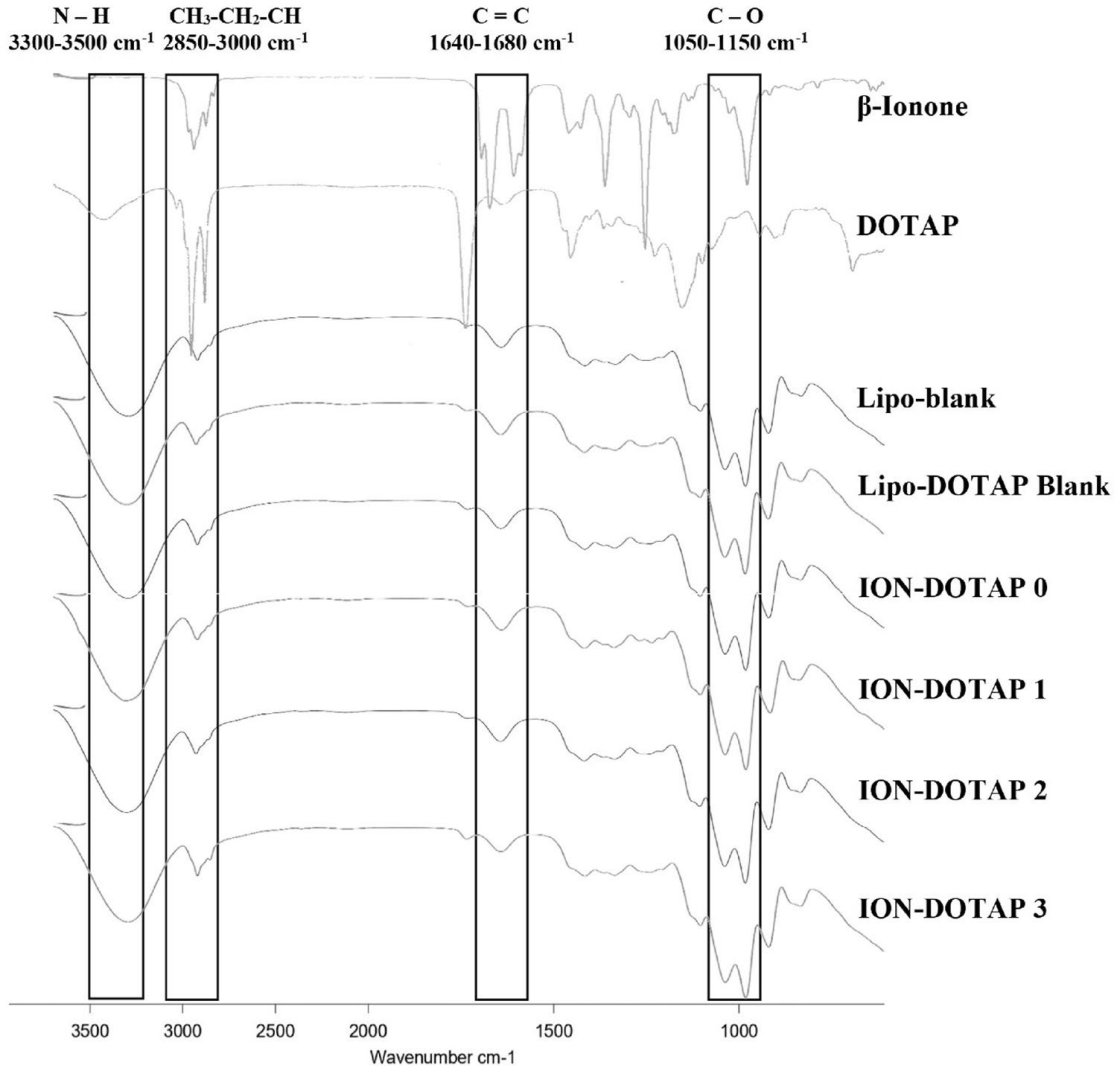
FTIR spectra of β-ionone, DOTAP, , ION-DOTAP liposomes, and Lipo-DOTAP Blank, ION-DOTAP 1, ION DOTAP 2, ION DOTAP 3 Lipo-Blank contained no DOTAP or β-ionone, while Lipo-DOTAP 3contained DOTAP at the highest concentration without the addition of β-ionone.

Identification of the compound’s structure was completed using the FTIR Alpha II Spectrophotometer within the range of wave numbers 4000–600 cm^-1^. The spectra of liposome constituents can be seen in Fig. 3, while those of the liposome formula produced can be seen in Fig. 4.

In the FTIR analysis, the Lipo-blank liposome spectra showed absorption bands identical to those in the spectra of the liposome constituents, as shown in Fig. 3. However, a decrease in the intensity of the absorption band after liposome formation occurred. The observed bands of the N–H functional group (3300–3500 cm^−1^) were identical to the DOTAP absorption band, the C = C group band (1640–1680 cm^−1^) was identical to the β-ionone absorption band, and the C–O group band (1050–1150 cm^−1^) was identical to the absorption band of β-ionone, DSPE-mPEG_2000_, and cholesterol. The CH_3_–CH_2_–CH group (2850–3000 cm^−1^) was identical to the absorption band of all the constituents of liposomes.

According to the overlaid spectra of β-ionone liposomes prepared with various concentration of DOTAP shown in Fig. 4, there were observed bands of N–H, CH_3_–CH_2_–CH, C = C (alkene), dan C–O functional groups for all formula. There are disappearances of some identical peaks of β-ionone and DOTAP observed for β-ionone liposomes with DOTAP addition. The spectra profiles of β-ionone liposomes show increasing the intensity and widening of absorption band of N–H groups that represent the DOTAP. However, spectra shown for each β-ionone liposomes showed no significant differences in the intensity of same type of absorption band.

### Differential thermal analysis (DTA) of β-ionone liposomes

**Figure 5:**
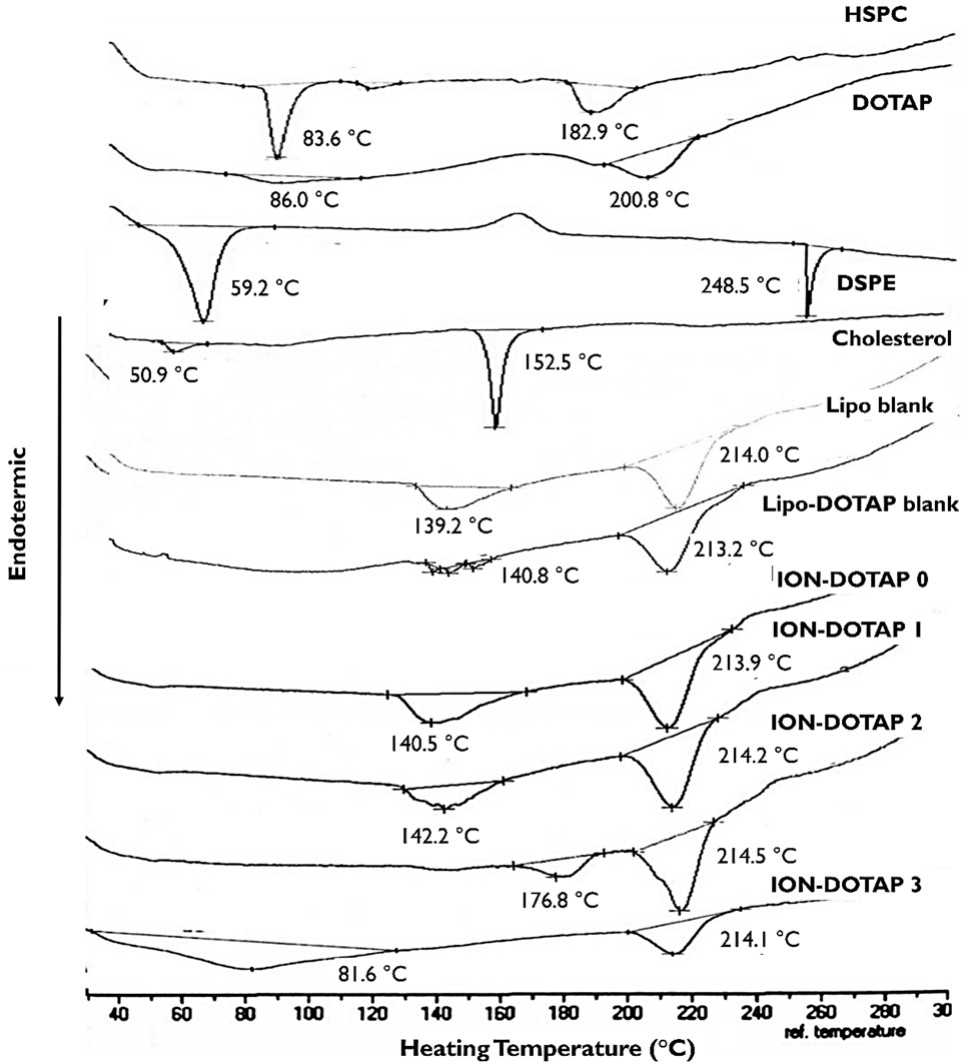
Thermogram profile of HSPC, DOTAP, DSPE-mPEG2000, Cholesterol, Lipo-Blank, Lipo-DOTAP Blank, ION-DOTAP-0, ION-DOTAP-1, ION-DOTAP-2, and ION-DOTAP-3.

The thermal analysis results produced using differential thermal analysis (DTA) on each liposome component and liposome formula can be seen in the thermogram shown in Fig. 5. The results show that the DTA thermogram of liposome constituents, i.e., DOTAP, experienced endothermic peaks at 86.0 °C and 200.8 °C, while the HSPC thermogram registered two endothermic peaks at 83.6 °C and 182 °C. On the other hand, the DSPE-mPEG2000 thermogram recorded endothermic peaks at 59.2 °C and 249.2 °C, and cholesterol at 50.9 °C and 152.5 °C. The Lipo-blank, which consisted of a mixture of cholesterol, HSPC, and DSPE-mPEG2000, registered two endothermic peaks at 139.2 °C and 214.0 °C. The formation of liposomes produced endothermic peak shifts.

Figure 5 also contains the results of the overlaid DTA thermograms of the liposomes, namely: DOTAP, Lipo-blank, Lipo-DOTAP Blank, ION-DOTAP-0, ION-DOTAP-1, ION-DOTAP-2, and ION-DOTAP-3. Each formula had two endothermic peaks. For example, thermograms on Lipo-DOTAP Blank, ION-DOTAP-0, and ION-DOTAP-1 liposomes produced endothermic peaks of approximately 140.2–140.8 °C and 213.2–214.2 °C. The endothermic peak formed was identical to the peaks of its constituents, namely: cholesterol and DOTAP.

Furthermore, the thermogram of ION-DOTAP 2 liposomes underwent a shift with endothermic peaks at temperatures of 176 °C and 114 °C. Meanwhile, the ION-DOTAP-3 liposomes, which contain the highest concentration of DOTAP, experienced a significant shift of endothermic peaks at 81.6 °C and 214.1 °C, with relatively wide peaks when compared to the those of the other liposomes. It also resulted in the loss of the endothermic peak of 152.5 °C, which is identical to that of cholesterol. This result confirms that the ION-DOTAP-3 thermogram experienced an endothermic peak increasingly similar to that of DOTAP.

### The calcein released from β-ionone liposomes

**Figure 6:**
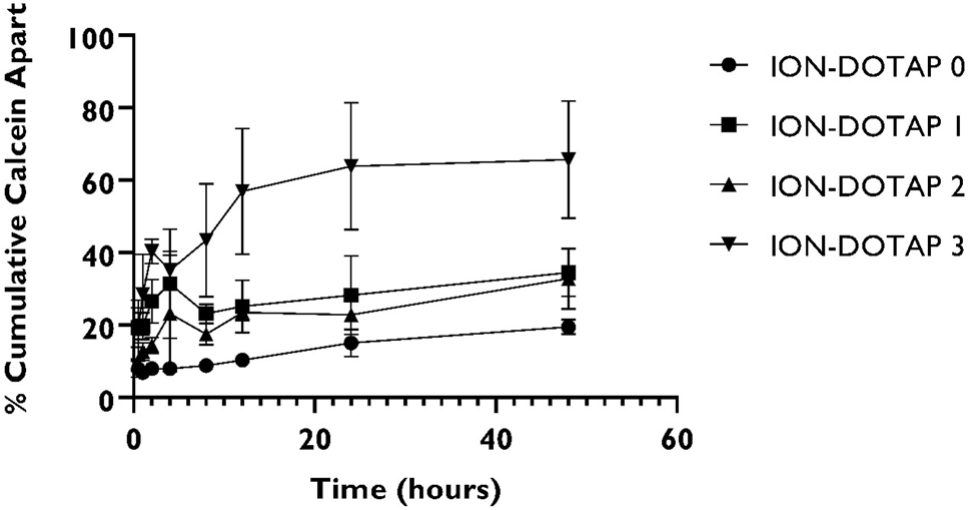
The calcein release profile of ION-DOTAP-0, ION-DOTAP-1, ION-DOTAP-2, and ION-DOTAP-3 liposomes in PBS pH 7.4 as the release media at a temperature of 37 °C.

Calculation of the cumulative percentage of the calcein released from the liposomes indicates that the smallest percentage of the calcein released was in the ION-DOTAP-0, indicating that this liposome has the most rigid structure compared to those of other formulas as presented in Fig. 6. Meanwhile, the highest percentage of released calcein was observed in ION-DOTAP 3 liposomes.

In addition, the release flux value obtained from this study reveals that the liposome ION-DOTAP-3 had the largest flux value (6.7074** ± **1.8650), followed by ION-DOTAP-2 (2.8357 ± 0.7453), ION-DOTAP 1 (1.6169 ± 0.4700) and ION-DOTAP-0 (0.2674 ± 0.0545) as shown in Table 1.

**Table 1:** The release flux value of calcein released from β-ionone liposomes prepared with various concentrations of added DOTAP.

Formula	Calcein release flux (μg/cm^2^/h, n = 3)
ION-DOTAP 0	0.2674 ± 0.0545
ION-DOTAP 1	1.6169 ± 0.4700
ION-DOTAP 2	2.8357 ± 0.7453
ION-DOTAP 3	6.7074 ± 1.8650

### The cellular uptake of β-ionone liposomes in HeLa cells

**Figure 7:**
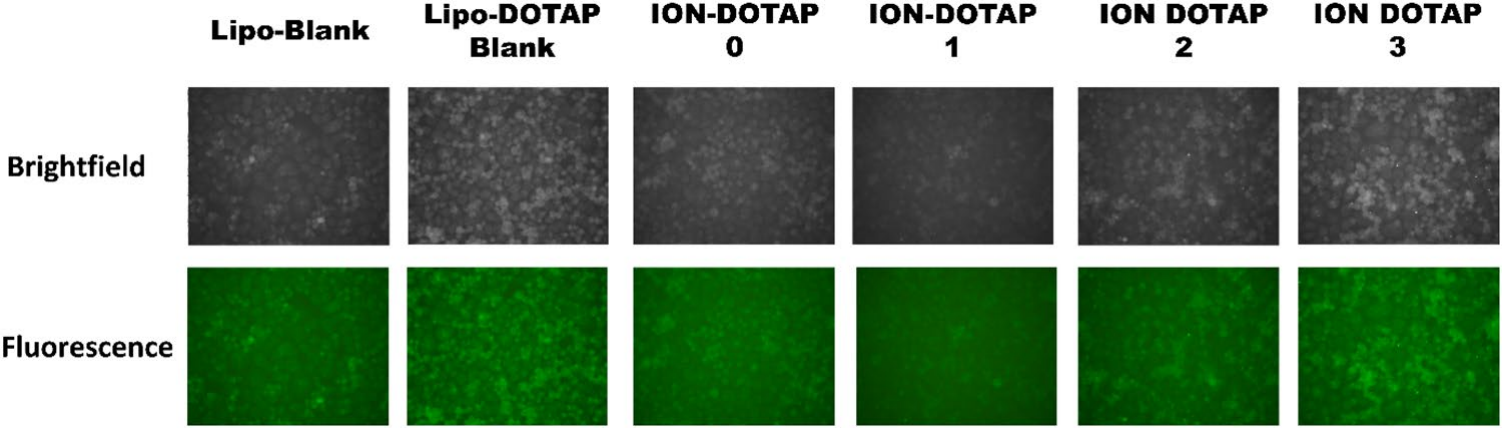
Photomicroscopy of cellular uptake of HeLa cells after incubation with β-ionone liposomes containing Coumarin-6 (in green color) prepared with various concentrations of DOTAP for two hours at 20 × magnification under a fluorescence microscope.

In Fig. 7, cells in the LIPO-blank treatment can be observed where the fluorescence intensity of coumarin-6 loaded into liposomes was relatively low. The liposomes containing coumarin-6 with high DOTAP levels increased the fluorescence intensity. Figure 7 shows that ION-DOTAP-0 liposomes containing coumarin-6 infiltrated the HeLa cells indicated by green fluorescence, while ION-DOTAP-1 liposomes were only slightly fluorescent. However, they were less visible and the fluorescence of the ION-DOTAP-2 liposomes was relatively weak in intensity as shown in the cell membrane and cytoplasm.

**Figure 8:**
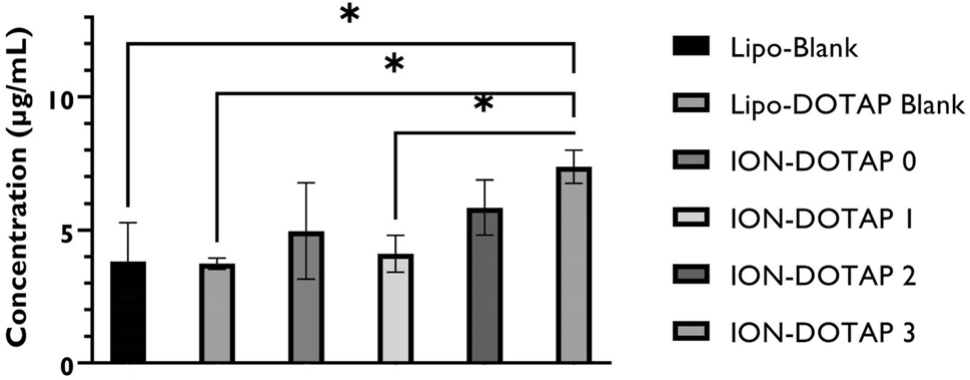
Coumarin-6 levels in HeLa cells post-incubation with β-ionone liposomes containing Coumarin-6 prepared with various concentrations of DOTAP for two hours.

Meanwhile, as shown in Fig. 8, the ION-DOTAP-3 liposome with the highest DOTAP content increased the fluorescence intensity and produced the highest cellular coumarin-6 levels than other formulas.

### In vitro cytotoxicity evaluation of β-ionone liposomes

**Figure 9:**
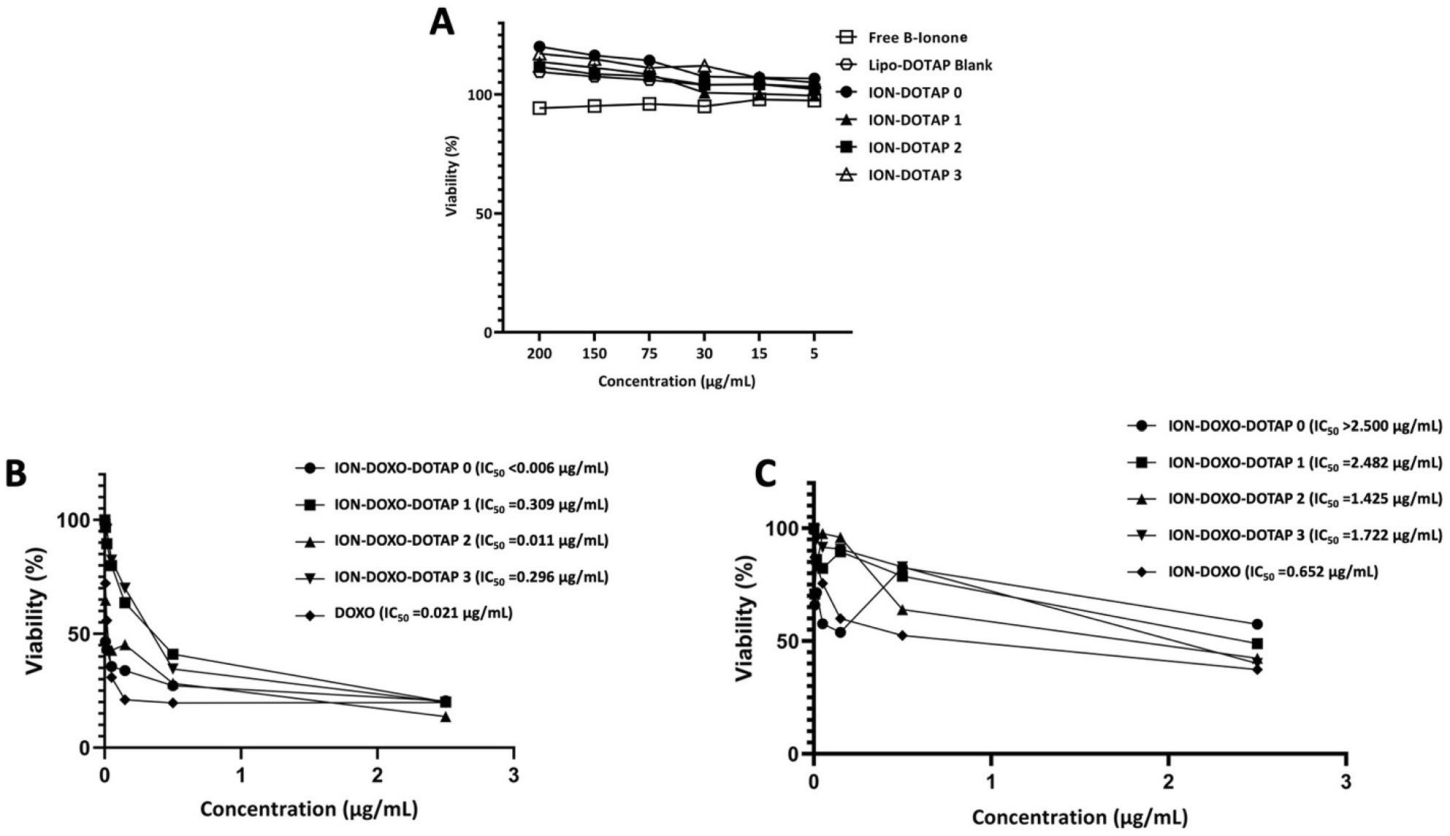
The viability of (**A**) HeLa cells after incubation of β-ionone liposomes and (**B**) T47D and (**C**) HeLa cells after incubation with β-ionone liposomes co-loaded with doxorubicin for 48 h.

The IC_50_ cytotoxicity test on HeLa cells aims to determine the effect of adding DOTAP to β-ionone liposomes in cancer therapy. The cells incubated with β-ionone liposomes for 48 h resulted in the percentage of cell viability shown in Fig. 9A. It can be seen that HeLa cells were not sensitive to β-ionone liposomes, and all treatments resulted in high cell viability.

To further evaluate the cytotoxic effects on these cells, DOXO was co-loaded into β-ionone liposomes. As shown in Fig. 9B, an increase in the cytotoxic effect also occurred in T47D cells, where the ION-DOXO-DOTAP-2 formula gave the lowest IC_50_ value compared to other liposome formulas, indicating its high cytotoxicity. On the other hand, ION-DOXO-DOTAP-0, which does not contain DOTAP, produces the smallest IC_50_ value. The results also showed that there is an increase in cytotoxicity of the ION-DOXO-DOTAP-2 formula in HeLa cells; however, ION-DOXO-DOTAP-0 produces the highest IC_50_ value (Fig. 9C).

## Discussion

In this study, liposomes were prepared to deliver β-ionone, a low water soluble and poorly permeable drug, used in anticancer treament^6,7^. However, the composition of phospholipids significantly affects liposomes' characteristics which may relate to their pharmacological activity. Therefore, this study evaluated the effect of the addition of DOTAP on the physical characteristics of β-ionone-loaded liposomes, including: particle size, zeta potential, morphology, infrared (IR) spectroscopic profiles, thermal analysis, and calcein release.

The addition of DOTAP affects the physicochemical characteristics of the liposomes, with increased DOTAP concentration resulting in larger particle size. This is probably due to the HSPC, cholesterol, and DSPE-mPEG2000 components which have ordered molecule arrangements producing a rigid membrane structure^36^. Moreover, the possibility exists of interaction between the polar groups of DOTAP and the choline groups of HSPC, both of which are positively charged and therefore repel each other, leading to a widening of the bilayer membrane’s space.

The spectra of liposomes loaded β-ionone with DOTAP addition experienced a reduction in the intensity and widening of the absorption band in the N–H group which is characteristic of DOTAP. The N–H group in DOTAP is the polar section of the phospholipid head^37^. Furthermore, the analysis of the absorption band on the alkyl group (CH_2_) can be used as an indicator of the regularity of the lipid chain arrangement. The reduced intensity and widening of the absorption band indicate an increase in the gauche conformation^38^. The spectra of liposomes formed showed a decrease in the intensity of the absorption band on the alkyl group, indicating a potential increase in the gauche conformation, which changed the composition of the lipid chain thereby rendering it more fluid.

Several identical peaks in DOTAP and β-ionone were not visible in the spectra of the liposomes loaded β-ionone with DOTAP addition, indicating that there could be physical interaction^39^. Meanwhile, the spectra shown for each liposome formula did not indicate a significant difference in the intensity of the absorption bands.

FTIR analysis, in determining the interaction of DOTAP with liposome membranes, can be performed by observing the absorption band on certain functional groups, namely: the phosphate group (R–PO_2_–R') and the ester group (–CO–O) present on the head of HSPC which is polar^40^. Neither the Lipo-blank FTIR spectra nor the blank Lipo-DOTAP showed any absorption bands in the two functional groups. Therefore, it is thought that hydrogen bonding occurs in both the phosphate and phospholipid ester groups due to stretching of the absorption band^41^. Lipo-blank and Lipo-DOTAP Blank liposomes did not show a significant shift in the absorption band and no new band formation, leading to the conclusion that only chemical interaction and no physical interaction occurs. Moreover, the ION-DOTAP-3 thermogram shows an endothermic peak that is increasingly similar to that of DOTAP. The widening and disappearance of the endothermic peaks on the thermogram indicate a change in the regularity of the lipid structure lipid^42^. In other words, adding DOTAP can reduce the rigidity of the membrane, thereby rendering it more fluid.

The calcein release test showed that the liposome ION-DOTAP-3 experienced the highest calcein release compared to the other formulas. The release of calcein from the liposomes increased in line with that of membrane fluidity^33^. It can therefore be concluded that ION-DOTAP 3 demonstrates the highest membrane fluidity. Based on the results of the analysis conducted to determine the physical characteristics of β-ionone liposomes, the addition of DOTAP can increase the particle size, rendering the zeta potential positive with the morphology of liposomes appearing spherical with a layered membrane. Furthermore, the results of thermogram data and spectroscopic profiles, show that the addition of DOTAP can cause the composition of the bilayer membrane to become more fluid. This result is in line with the calcein release profile which indicates that adding DOTAP can increase the fluidity of the liposome membrane.

With regard to the surface charge of liposomes, the liposome constituents such as HSPC and DSPE-mPEG_2000_ have a neutral charge, while β-ionone and cholesterol have no charge or are non-ionic^43^. Meanwhile, DOTAP is a positively charged phospholipid with an NH^4+^ group on the head of the phospholipid^19^. Consequently, adding DOTAP to liposomes increased the positivity of the potential charge. Increasing the concentration of DOTAP heightened the positive charge of the liposomes.

A cellular uptake test was carried out to determine the ability of β-ionone liposomes to infiltrate the HeLa cells. The levels of liposomes entering HeLa cells were calculated as coumarin-6 levels. Coumarin-6 is a hydrophobic compound trapped in the bilayer membrane and the measured coumarin-6 level indicates how many liposomes enter the cell. The green fluorescence indicates that β-ionone liposomes containing coumarin-6 are present in the cells^44^. The more intense the fluorescence color, the greater the amount of coumarin-6 absorbed^45^. Based on the findings of the quantification analysis of coumarin-6 levels, the ION-DOTAP-3 formula produced higher levels than other formulas. It is because the ION-DOTAP-3 liposomes contain cationic lipid (DOTAP) at a higher concentration than other formulas, resulting in a significant ionic interaction between negatively charged cancer cells and β-ionone liposomes containing DOTAP (cationic lipid)^46^.

It was also reported that particle size affects the endocytosis pathway of liposomes into cells. In this study, rising levels of DOTAP increased the particle size of liposomes, although this was in the range of < 200 nm which was still relatively modest. Consequently, no significant effect on cellular uptake was regarded as having occurred. The entry of β-ionone liposomes was due to the addition of DOTAP to cells through a process of endocytosis which requires energy to introduce foreign particles into the cell. The results demonstrated a significant difference between the ION-DOTAP-3 and the blank lipid formula, blank ION-DOTAP, and ION-DOTAP-1. In this study, it can be seen that, with the addition of higher DOTAP levels, β-ionone liposomes can increase cell uptake in HeLa cells. This greater cell uptake leading to increased coumarin-6 levels and microscopic observations of HeLa cells showed that the intensity of coumarin-6 rose with the occurrence of higher DOTAP levels.

The IC_50_ cytotoxicity test was carried out on HeLa cells to determine the effect of adding DOTAP to β-ionone liposomes in cancer therapy. Unfortunately, β-ionone liposomes had no cytotoxic effects even though the cellular uptake test results show that liposomes entered the cells. This result indicates that it is possible that these cells are not sensitive to β-ionone, which becomes the limitation of our study. Therefore, a cytotoxicity test was carried out by co-encapsulating DOXO into β-ionone liposomes (ION-DOXO-DOTAP liposomes). DOXO has been known as an anticancer compound sensitive to HeLa cells and T47D cells. The results showed that the ION-DOXO-DOTAP liposomes had toxicity to both cancer cells. The ION-DOXO-DOTAP-2 had the highest cytotoxicity. Compared to the ION-DOXO-DOTAP-0, which had no DOTAP addition, the toxicity is relatively similar to that of DOXO. This is related to liposome formulations with the addition of DOTAP providing better retention than liposomes without DOTAP. The increased drug release was proportional to the cell uptake, which also increased with the addition of DOTAP. This result is in line with the study of Lei et al. (2011), where it was reported that cytotoxicity results were associated with cellular uptake. Therefore, high cellular uptake will give an increased cytotoxicity value^47^.

## Conclusions

The results of this study indicate that the addition of DOTAP to β-ionone liposomes increased the particle size slightly and rendered the charge of the liposomes positive. In addition, the higher the DOTAP concentration added, the more fluid the liposomal membrane as indicated by the reduction in membrane crystallinity which supported the higher calcein release from the liposomes. The introduction of DOTAP into liposomes highly affects the physical properties of β-ionone liposomes, thereby increasing the cellular uptake and cytotoxicity. Proposing the incorporation of DOTAP into β-ionone liposomes requires further studies on its anticancer efficacy. From the results of this analysis, although the addition of DOTAP renders the liposomes more fluid, its ability to enhance the cellular uptake provides potential benefits when used as a delivery system for cancer therapy.

## Materials and methods

### Materials

In this study, β-ionone was purchased from Sigma-Aldrich Inc. (Saint Louis, USA). The liposome component was 1,2-dioleoyl-3-trimethylammonium propane (DOTAP), Hydrogenated Soybean Phosphatidylcholine (HSPC), and Distearoyl-glycerophosphoethanolamine–N–(Carbonyl-methoxy polyethylene glycol 2000 (DSPE-mPEG_2000_), which were products of Nof Corporation (Tokyo, Japan). Cholesterol was obtained from Wako Inc., Ltd. (Osaka, Japan), while calcein (Fluorexone^®^) was a product of Nacalai Tesque Inc. (Kyoto, Japan). The buffer components include phosphate buffer pH 7.4, KH_2_PO_4_, Na_2_HPO_4_, NaCl, and KCl were purchased from Merck Inc. (Darmstadt, Germany). Chloroform and methanol were products of Merck Inc. (Darmstadt, Germany). Other reagents and materials used were of non-technical grade.

### Preparation of β-ionone liposomes

β-ionone liposomes were prepared by the thin film hydration method. First, β-ionone, DOTAP, HSPC, DSPE-mPEG_2000_, and cholesterol were dissolved in chloroform. As shown in Table 2, appropriate amounts of each solution were, mixed in a round bottom flask. The chloroform was then evaporated and completely removed using a rotary evaporator at 55 °C to form a thin film layer on the flask wall. The thin film formed was hydrated with phosphate-buffered saline (PBS) pH 7.4 and sonicated using a sonicator at 55 °C for 10 min^27^. Extrusion was subsequently carried out with an extruder (Avanti^®^, Mini Extruder, Avanti Polar Lipid Inc., Alabama, USA) by passing liposomes through a polycarbonate membrane with various pore sizes, i.e., 400, 200, and 100 nm, with a heating block at a temperature of 55–60 °C to produce liposomes approximately 100 nm in size^42^.

**Table 2:** Composition of β-ionone liposomes.

Components	Composition (mole ratio)					
	Lipo-blank	Lipo-DOTAP blank	ION-DOTAP-0	ION-DOTAP-1	ION-DOTAP-2	ION-DOTAP-3
β-ionone	–	–	30	30	30	30
DOTAP	–	8	0	2.5	5	8
HSPC	35	35	35	35	35	35
Cholesterol	25	25	25	25	25	25
DSPE-mPEG_2000_	10	10	10	10	10	10
Hydrating buffer	Phosphate buffered saline (PBS) pH 7.4					

### Evaluation of physical characteristics of β-ionone liposomes

The particle size of β-ionone liposomes were determined by the dynamic light scattering method using a Delsa Nano C Particle Analyzer at 25 °C. Meanwhile, the zeta potential was measured using the electrophoresis light scattering method using the Malvern Zetasizer Nano Series Analyzer at 25 °C at the Sepuluh Nopember Institute of Technology (ITS), Surabaya, Indonesia. The liposomes were diluted with demineralized water, placed in a cuvette, and measured for particle size and zeta potential^27^.

### Evaluation of vesicle morphology of β-ionone liposomes using transmission electron microscopy (TEM)

Liposome morphology, determined using the JEOL JEM-1400 TEM (Jeol Inc., USA) instrument, was carried out at the Laboratory of Chemistry of Gadjah Mada University (UGM), Yogyakarta, Indonesia. Liposomes were first diluted using demineralized water at a volume ratio of 1:5 of liposome and water respectively. The sample was subsequently stained with 4% phosphotungstic acid at a ratio of 1:1. Samples were placed on a copper grid, and images taken at various magnification sizes^15^.

### Fourier-transform infrared spectroscopy (FTIR) Analysis of β-Ionone Liposomes

FTIR evaluation was carried out to determine molecular interactions within the liposomes. Before the analysis, each β-ionone liposome was hydrated with PBS pH 7.4 containing 7% sucrose and freeze dried. FTIR analysis was then conducted using an FTIR Alpha II Spectrophotometer (Bruker, Ettlingen, Germany). The sample was put into the sample container and the spectra measured at wave numbers of 4000–600 cm^-1^.

### Differential thermal analysis (DTA) of β-ionone liposomes

DTA is used to evaluate changes in sample properties evident from the endothermic and exothermic peaks on the DTA thermogram when heat is applied. The tests were carried out using a DTA instrument (Mettler Toledo FP90, Switzerland). Each β-ionone liposome was hydrated with PBS pH 7.4 containing 7% sucrose and freeze-dried before analysis. The freeze-dried samples were placed in aluminum crucibles and heated from 30 to 300 °C at the rate of 10 °C/min^27^.

### Evaluation of calcein release

#### Preparation of β-ionone liposomes containing calcein for release study

The β-ionone liposomes were prepared on the basis of the above method. The thin lipid film formed was then hydrated with 1 mL of PBS pH 5.0 containing 3.4 mM calcein (Nacalai Tesque Inc., Kyoto, Japan). Extrusion was subsequently carried out with an extruder (Avanti^®^, Mini Extruder, Avanti Polar Lipid Inc., Alabama, USA) by passing liposomes through a polycarbonate membrane with various pore sizes of 400, 200, and 100 nm with a heating block at 55–60°C^42^. The free calcein was subsequently separated from the liposomes by placing them into the Amicon^®^ Ultra-4 Centrifugal Filter Unit tube with a molecular weight cut-off (MWCO) of 10,000 Da (Merck Millipore, Darmstadt, Germany). The tube was centrifuged using a Hettich Rotofix 32 Benchtop Centrifuge (Hettich Lab., Tuttlingen, Germany) at 3500 rpm in order to remove the unentrapped or free calcein.

#### Calcein release assay from β-ionone liposomes

A calcein release study was conducted by dialysis. The liposomes were placed in the Spectra/Por^®^7 dialysis membrane with MWCO (Spectrum Laboratories, USA) of 3500 Da. 50 mL of PBS pH 7.4 was employed as a release medium with a stirring speed of 350 rpm at 37 °C. C. Sampling was performed at 0.5, 1, 2, 4, 8, 12, 24, and 48 h by replacing some samples with the same volume of fresh PBS pH 7.4. The calcein fluorescence intensity was subsequently measured using the GloMax^®^-Multi Detection System (Promega Corporation, Madison, USA) microplate reader at λ_ex_ = 475 nm and λ_em_ = 500–550 nm.

### The cellular uptake assay of β-ionone liposomes

#### Preparation of β-ionone liposomes containing coumarin-6 as the fluorescent labeling agent

The first procedure involved dissolving each liposome component in chloroform. Liposomes were prepared according to the formula in the Table 2 by adding 0.3 mg Coumarin-6 to each mL of liposomes containing 9.7 mg total lipid of HSPC, Cholesterol, and DSPE-mPEG_2000_. The amount of coumarin-6 added was in accordance with the previous study of Miatmoko et al. (2021), which successfully represented liposome uptake in cancer cells^48^. Each component solution was appropriately mixed in a round bottom flask covered with aluminum foil. The organic solvent was then placed in a rotary evaporator at a temperature of 55 °C until all organic solvents were removed and formed a thin film which was hydrated using 1 mL of PBS pH 7.4, vortexed, and sonicated for 10 mins at 55 °C. Liposomes were subsequently extruded using polycarbonate membranes of sizes from 400, 200, to 100 nm to produce liposomes with a size of about 100 nm.

#### The cellular uptake assay

Initially, the HeLa cells, which were obtained from Stem Cell Research and Development Center, Universitas Airlangga, were suspended in RPMI 1640 medium containing 10% Fetal Bovine Serum (FBS) and 1% Penicillin–Streptomycin antibiotic at a concentration of 1 × 10^6^ cells/mL, placed into a 6-well plate, and incubated at 37 °C with 5% CO_2_ environment for 24 h. Cells can be used for cellular uptake assay if they have a confluence of 70–80%^44^. The medium was replaced with β-ionone liposomes at a coumarin-6 concentration of 10 μg/mL and incubated for two hours, with the media being disposed of. The cells were first twice washed with PBS pH 7.4 and then added to 1 mL of methanol and left for 5–10 mins. After the methanol had been discarded, the cells were washed twice with PBS pH 7.4, containing 0.5% Tween 80 and approximately 1 mL of PBS pH 7.4 containing 1% FBS was added and left for 15 min. The cells were then washed twice with PBS solution pH 7.4. The final step was to observe the cells by means of a fluorescence microscope with an FITC filter^44^.

#### Measurement of coumarin-6 levels in HeLa cells

In the first step, the cells were prepared with the same procedure as that for the cellular uptake assay and the samples were incubated for two hours. The medium was then discarded and the cells washed twice with PBS pH 7.4. In order to measure the amount of intra-cellular coumarin-6, the cells were lysed with 100 μL of lysis buffer containing 10 mM NaCl, 1 mM Ethylenediaminetetraacetic acid (EDTA), and 0.5% Triton X-100 by means of pipetting and added to 900 μL PBS pH 7.4 to enable collection of the samples. The cell suspension was vortexed and centrifuged at 12,000 rpm for ten minutes. After centrifugation, the supernatant's fluorescence intensity was measured using GloMax^®^-Multi Detection System (Promega Corporation, Madison, USA) microplate reader at λ_ex_ = 475 nm and λ_em_ = 500–550 nm^44^.

#### In vitro cytotoxicity assay

##### Preparation of liposomes containing doxorubicin

In addition to β-ionone liposomes, we prepared β-ionone liposomes co-loaded with Doxorubicin (DOXO) to produce ION-DOXO-DOTAP liposomes and evaluate their cytotoxicities on T47D and HeLa cells. β-ionone liposomes co-loaded with DOXO were prepared by the same formula and methods as β-ionone liposomes; however, the 0.65 M Triethylamine was used as the hydrating solution. The liposomes were then eluted through a column packed with Sephadex G50 using PBS pH 7.4. DOXO was added at DOX/total lipid ratio of 1:5, respectively, and the mixture was then incubated in a water bath at 60 °C for 10 min. The liposomes were further separated from the free DOXO by elution through the Sephadex G50 gel column.

##### WST assay

The in vitro cytotoxicity test procedure was evaluated for T47D cells and HeLa cells using the WST-1 assay method. The T47D and HeLa cell were obtained from Stem Cell Research and Development Center, Universitas Airlangga, and then cultured in Roswell Park Memorial Institute (RPMI)-1640 medium containing 10% Fetal Bovine serum and 1% Penicillin Streptomycin in a humidified atmosphere at 37 °C with 5% CO_2_ air supply. For the assay, T47D and HeLa cells with a concentration of 5 × 10^3^ cells were added to each well of 96-well plates and incubated for 24 h before treatment.

To evaluate the cytotoxicity, β-ionone liposomes and β -ionone liposomes co-loaded with DOXO were prepared at various concentrations. These samples were added to each well containing the cells and incubated for 48 h. After the treatment, the cell number was determined using WST-1 assay. The cell viability was determined as the relative measurement to the untreated cells by evaluating the absorbance of each well at 440 nm using a microplate reader. The IC_50_ values of the compounds were calculated using the percentage of living cells and analyzed using SPSS (probit) software.

### Data analysis

The data for analysis consisted of the average ± standard deviation measured in replications. First, a Kolmogorov–Smirnov test was used to analyze the numerical data s for normal distribution. If the data was normal (*P* value ≥ 0.05), the quantitative analysis was analyzed using One Way Analysis of Variance. If the *P* value < 0.05, the data analysis was followed by a post hoc test incorporating the use of Honestly Significant Difference (HSD). The analysis was conducted using the IBM SPSS Statistics software version 24.

## Data Availability

The datasets used and/or analyzed during the current study available from the corresponding author on reasonable request.

## References

[1] Ansari M, Emami S (2016). β-Ionone and its analogs as promising anticancer agents. Eur. J. Med. Chem..

[2] Scolastici C, de Conti A, Cardozo MT, Ong TP, Purgatto E, Horst MA, Heidor R, Furtado KS, Bassoli BK, Moreno FS (2015). β-ionone inhibits persistent preneoplastic lesions during the early promotion phase of rat hepatocarcinogenes: TGF-α, NF-κB, and p53 as cellular targets. Nutr. Cancer.

[3] Lee SM (2013). Anti-proliferative effects of β-ionone on human lung cancer A-549 cells. J. Life Sci..

[4] Sharma V (2014). β-Ionone derived apoptosis inducing endoperoxides; Discovery of potent leads for anticancer agents. Eur. J. Med. Chem..

[5] Dong H-W (2019). Beta-ionone-inhibited proliferation of breast cancer cells by inhibited COX-2 activity. Arch. Toxicol..

[6] Lide, D. R. et al.* CRC Handbook of Chemistry and Physics*. (Taylor and Francis Group LLC, 2009).

[7] OECD. Oecd sids Β-ionone. pp. 4–5 (2004).

[8] Moslehi M (2020). Preparation, optimization and characterization of chitosancoated liposomes for solubility enhancement of furosemide: A model BCS IV drug. Iran. J. Pharm. Res..

[9] Skupin-Mrugalska, P. *Liposome-Based Drug Delivery for Lung Cancer*. *Nanotechnology-Based Targeted Drug Delivery Systems for Lung Cancer* (Elsevier Inc., 2019) 10.1016/b978-0-12-815720-6.00006-x.

[10] Laouini A (2012). Preparation, characterization and applications of liposomes: State of the art. J. Colloid Sci. Biotechnol..

[11] Patel GM, Shelat PK, Lalwani AN (2017). QbD based development of proliposome of lopinavir for improved oral bioavailability. Eur. J. Pharm. Sci..

[12] Strieth S (2008). Paclitaxel encapsulated in cationic liposomes increases tumor microvessel leakiness and improves therapeutic efficacy in combination with cisplatin. Clin. Cancer Res..

[13] Jufri M (2004). Arah dan perkembangan liposome drugs delivery systems. MIK.

[14] Kawakami S, Suzuki S, Yamashita F, Hashida M (2006). Induction of apoptosis in A549 human lung cancer cells by all-trans retinoic acid incorporated in DOTAP/cholesterol liposomes. J. Control. Release.

[15] Wieber A, Selzer T, Kreuter J (2012). Physico-chemical characterisation of cationic DOTAP liposomes as drug delivery system for a hydrophilic decapeptide before and after freeze-drying. Eur. J. Pharm. Biopharm..

[16] Ito I (2003). Increased uptake of liposomal-DNA complexes by lung metastases following intravenous administration. Mol. Ther..

[17] Tao J (2016). Optimization of a cationic liposome-based gene delivery system for the application of miR-145 in anticancer therapeutics. Int. J. Mol. Med..

[18] Hattori Y, Tamaki K, Ozaki K, Ichi Kawano K, Onishi H (2019). Optimized combination of cationic lipids and neutral helper lipids in cationic liposomes for siRNA delivery into the lung by intravenous injection of siRNA lipoplexes. J. Drug Deliv. Sci. Technol..

[19] Gascón AR, Pedraz JL (2008). Cationic lipids as gene transfer agents: A patent review. Expert Opin. Ther. Pat..

[20] Chang SF, Yeh CC, Chen PJ, Chang HI (2018). The impact of lipid types and liposomal formulations on osteoblast adiposity and mineralization. Molecules.

[21] Choi, S. Precise control of liposome size using characteristic time depends on solvent type and membrane properties. **18**, 1–19 (2022) 10.21203/rs.3.rs-2162076/v1. 10.1038/s41598-023-31895-z PMC10036480 36959258

[22] Nchinda G, Überla K, Zschörnig O (2002). Characterization of cationic lipid DNA transfection complexes differing in susceptibility to serum inhibition. BMC Biotechnol..

[23] Campbell RB, Balasubramanian SV, Straubinger RM (2001). Phospholipid-cationic lipid interactions: Influences on membrane and vesicle properties. Biochim. Biophys. Acta Biomembr..

[24] Lou G, Anderluzzi G, Woods S, Roberts CW, Perrie Y (2019). A novel microfluidic-based approach to formulate size-tuneable large unilamellar cationic liposomes: Formulation, cellular uptake and biodistribution investigations. Eur. J. Pharm. Biopharm..

[25] Wang Q, Rojas EC, Papadopoulos KD (2012). Cationic liposomes in double emulsions for controlled release. J. Colloid Interface Sci..

[26] Takechi-Haraya Y (2016). Atomic force microscopic analysis of the effect of lipid composition on liposome membrane rigidity. Langmuir.

[27] Chen L (2018). Characterizations on the stability and release properties of β-ionone loaded thermosensitive liposomes (TSLs). J. Agric. Food Chem..

[28] Barroso RP, Basso LGM, Costa-Filho AJ (2015). Interactions of the antimalarial amodiaquine with lipid model membranes. Chem. Phys. Lipids.

[29] Basso LGM, Rodrigues RZ, Naal RMZG, Costa-Filho AJ (2011). Effects of the antimalarial drug primaquine on the dynamic structure of lipid model membranes. Biochim. Biophys. Acta Biomembr..

[30] Miatmoko A (2019). Dual loading of primaquine and chloroquine into liposome. Eur. Pharm. J..

[31] Miatmoko A (2021). Interactions of primaquine and chloroquine with PEGylated phosphatidylcholine liposomes. Sci. Rep..

[32] Ghosh AK, Basu R, Nandy P (1995). Lipid perturbation of liposomal membrane of dipalmitoyl phosphatidylcholine by chloroquine sulphate—A fluorescence anisotropic study. Colloids Surf. B Biointerfaces.

[33] Maherani B, Arab-Tehrany E, Kheirolomoom A, Geny D, Linder M (2013). Calcein release behavior from liposomal bilayer; Influence of physicochemical/mechanical/structural properties of lipids. Biochimie.

[34] Hara M (2008). Stabilization of liposomal membranes by carotenoids: Zeaxanthin, zeaxanthin glucoside and thermozeaxanthin. Mater. Sci. Eng. C.

[35] Danaei M (2018). Impact of particle size and polydispersity index on the clinical applications of lipidic nanocarrier systems. Pharmaceutics.

[36] Barenholz Y (2012). Doxil^®^—The first FDA-approved nano-drug: Lessons learned. J. Control. Release.

[37] Juszkiewicz K, Sikorski AF, Czogalla A (2020). Building blocks to design liposomal delivery systems. Int. J. Mol. Sci..

[38] Refai H, Hassan D, Abdelmonem R (2017). Development and characterization of polymer-coated liposomes for vaginal delivery of sildenafil citrate. Drug Deliv..

[39] Kesharwani P, Md S, Alhakamy NA, Hosny KM, Haque A (2021). Qbd enabled azacitidine loaded liposomal nanoformulation and its in vitro evaluation. Polymers Basel..

[40] Cieślik-Boczula K (2012). Interaction of quercetin, genistein and its derivatives with lipid bilayers—An ATR IR-spectroscopic study. Vib. Spectrosc..

[41] Ezer N, Sahin I, Kazanci N (2017). Vibrational spectroscopy alliin interacts with DMPC model membranes to modify the membrane dynamics: FTIR and DSC Studies. Vib. Spectrosc..

[42] Miatmoko A (2021). Interactions of primaquine and chloroquine with PEGylated phosphatidylcholine liposomes. Sci. Rep..

[43] Aramaki K (2016). Charge boosting effect of cholesterol on cationic liposomes. Colloids Surf. A Physicochem. Eng. Asp..

[44] Sun Y (2017). Cellular uptake mechanism and clearance kinetics of fluorescence-labeled glycyrrhetinic acid and glycyrrhetinic acid–modified liposome in hepatocellular carcinoma cells. Environ. Toxicol. Pharmacol..

[45] Muthu MS, Kulkarni SA, Xiong J, Feng SS (2011). Vitamin E TPGS coated liposomes enhanced cellular uptake and cytotoxicity of docetaxel in brain cancer cells. Int. J. Pharm..

[46] Smith MC, Crist RM, Clogston JD, McNeil SE (2017). Zeta potential: A case study of cationic, anionic, and neutral liposomes. Anal. Bioanal. Chem..

[47] Lei T (2011). Comparing cellular uptake and cytotoxicity of targeted drug carriers in cancer cell lines with different drug resistance mechanisms. NIH Public Acess.

[48] Miatmoko, A. et al. The effect of chitosan addition on cellular uptake and cytotoxicity of ursolic acid niosomes. *An. Acad. Bras. Cienc.* **93**, (2021) 10.1590/0001-3765202120201850. 10.1590/0001-3765202120201850 34287462

